# Case Report: Adult Onset Still’s Disease after vaccination against Covid-19

**DOI:** 10.12688/wellcomeopenres.17345.1

**Published:** 2021-12-07

**Authors:** Ujjwol Risal, Anup Subedee, Raju Pangeni, Rakshya Pandey, Suravi Pandey, Sudeep Adhikari, Buddha Basnyat

**Affiliations:** 1 Internal Medicine, Hospital for Advanced Medicine and Surgery, Kathmandu, Nepal; 2 Pulmonary and Critical Care, Hospital for Advanced Medicine and Surgery, Kathmandu, Nepal; 3 Internal Medicine, Pyuthan Hospital, Pyuthan, Nepal; 4 Oxford University Clinical Research Unit, Patan Hospital, Kathmandu, Nepal

**Keywords:** Covid-19 vaccination, AOSD

## Abstract

Vaccination against the virus responsible for COVID-19 has become a key in preventing mortality and morbidity related to the infection. Studies have shown that the benefits of vaccination outweigh the risks. However, there are concerns regarding serious adverse events of some vaccines,      although they are fortunately      rare. Here, we report a case of a 47-year-old female from Kathmandu who presented with high grade fever, dry cough and erythematous rash a week after exposure to the Oxford-AstraZeneca vaccine. She had hepatosplenomegaly, persistent leucocytosis, anaemia and thrombocytosis along with markedly raised inflammatory markers. Her tests for infectious causes and haematological malignancies was negative and she showed no response to multiple antibiotics. Finally, she had a dramatic response to steroids with disappearance of fever and normalization of other laboratory parameters. Hence, she was diagnosed with       Adult-onset Still’s      Disease (AOSD). She was under methotrexate and prednisolone tapering dose and doing well as at time of writing. The trigger for the disease was hypothesized to be the vaccine because of the strong temporal association.

## Introduction

 COVID-19 is a major pandemic currently affecting the whole world, and Nepal is noexception. As of 15 September 2021, a total of 773,529 people had been infected and 10,889 people have already lost their lives in Nepal ^1^. However, various vaccines have been tested and made available which are highly effective in preventing both the morbidity and mortality associated with the disease ^2^. However, there are concerns regarding adverse events following vaccination ^3^. Adult-onset Still’s Disease (AOSD) is a multisystem autoinflammatory disease characterized by high grade fever, inflammatory arthritis, and an evanescent rash. It is associated with profound systemic inflammation marked by high inflammatory markers, leucocytosis and high ferritin levels. The diagnosis is made clinically after ruling out other aetiologies ^4^. We describe a case of AOSD following administration of the Oxford-AstraZeneca vaccine in Nepal. AOSD is a rare autoinflammatory disease and diagnosis of this case itself is a challenge, especially in a country like Nepal, where rheumatology is a budding specialty. This case were follows only two other reported similar cases after vaccination against COVID-19 ^5, 6^.

## Case presentation

**Figure 1.**
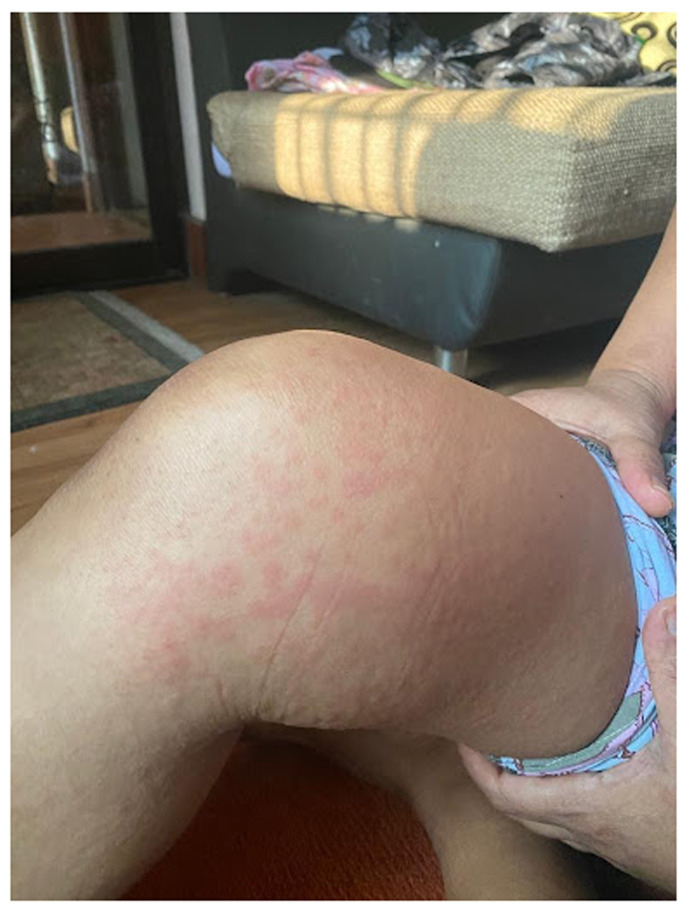
Erythematous macular rash involving right thigh of the patient.

A 47-year-old female from Kathmandu, Nepal, non-smoker presented in April 2021 to the out-patient department of a tertiary hospital in Kathmandu, with a history of intermittent fever and sore throat for nine days. After three days of fever, she developed itchy erythematous rashes bilaterally involving thighs, legs and hands, especially during the spiking of fever. She had received the first dose of the Oxford-AstraZeneca vaccine seven days prior to the onset of fever. She had no notable past medical and family history. When she presented to us, she was already on two antibiotics, namely levofloxacin 750 mg and azithromycin 500 mg, once daily for a couple of days from a local medical shop. Upon examination, her temperature was 38.55 ^0^ Celsius, and had erythematous rashes over bilateral legs and hands ( Figure 1). The remaining systemic examination found no further anomalies. SARS-CoV-2 polymerase chain reaction (PCR) was negative. The initial laboratory investigations ( Table 1) showed anaemia with neutrophilic leuc ocytosis and thrombocytosis, raised C-reactive protein (CRP), markedly raised ferritin, and mild transaminitis. Ultrasound of the abdomen showed hepatosplenomegaly. She was admitted with intravenous ceftriaxone 2 g once daily and oral doxycycline 100 mg twice daily. Blood and urine cultures were performed, which later revealed no growth. Serological tests for dengue, scrub typhus, leptospirosis and malaria were negative. There was no response to antibiotics after 72 hours. Further investigations were performed considering possible non-infectious causes of fever. Her antinuclear antibody (ANA) was weakly positive with a fine speckled pattern. Rheumatoid factor, anti-citrullinated peptide antibodies (ACPA), and anti-neutrophil cytoplasmic antibodies (ANCA) were negative. Her histopathological bone marrow examination was normal. On the fourth day of her admission, she also developed pain and swelling of her left elbow. On examination, the elbow joint was warm and tender with restriction of movement. She was then started on prednisolone at 1 mg/kg (60 mg) once daily, with continued antibiotics. After starting prednisolone, she had no further episodes of fever. Her joint pain improved, and she experienced no further rash. The final diagnosis of AOSD was made based on criteria defined by Yamaguchi ^7^. Methotrexate 7.5 mg per week was started. The antibiotics were stopped on the seventh day, and she was discharged with prednisolone and methotrexate. She is under regular follow-up for her condition as at time of writing this report. During her latest follow-up in September 2021, she was afebrile and had no other symptoms. She is currently receiving prednisolone 10 mg daily and methotrexate 15 mg per week as at time of writing.

**Table 1.** Laboratory investigations of the patient.

Laboratory parameters	Normal range	2021 April 2 (day of admission)	2021 April 4 (second day of admission)	2021 April 6 (fourth day- steroid started)	2021 April 7 (one day after starting steroid)	2021 September 9 (last follow-up)
Hemoglobin (gm/dl)	12.1- 15.1	9.3	9.2	9.8	9	12.5
White cell count(/mm ^3^)	4500- 11000	17050	18100	24300	16150	7700
Neutrophil percentage	55- 70	86	89	91	86	57
Platelets(/mm ^3^)	150000- 450000	471000	687000	1400000	956000	213000
C-Reactive Protein(mg/dL)	0.8- 1.0	150	>150	>150	108	0.8
Serum Ferritin(ng/ml)	24- 336	2914.6				
Aspartate transaminase(IU/L)	5- 40	218				28
Alanine transaminase(IU/L)	7- 55	194				73

## Discussion

Vaccination against COVID-19 has become the most effective way to curb the current pandemic and its effects on activities worldwide ^2^ As at time of writing this report, several vaccines had undergone trials and been given approval to be rolled out in several countries. Minor side effects after vaccination are fairly common; however, there have been some concerns regarding rare but serious adverse events of some vaccines ^3^. For example, cerebral venous sinus thrombosis has been described in some patients who have received the Ad26.COV2.S (Janssen/Johnson & Johnson) COVID-19 vaccine ^8^. Likewise, several cases of thrombosis have developed following vaccination with the Oxford-AstraZeneca vaccine ^9^. Autoimmune diseases themselves are rare, and those developing following vaccination are even rarer. Some cases of autoimmune diseases following COVID-19 vaccination have been described ^10, 11^.

Adult-onset Still's disease (AOSD) is a rare auto-inflammatory disorder characterized by a high spiking fever, arthralgia (with or without synovitis), maculo-papular salmon-pink evanescent skin rash, striking leucocytosis with neutrophilia ^4^. There is no definitive confirmatory test for AOSD, but there are a few sets of clinical criteria which are used in the diagnosis ^7^. In one study in France, the incidence of the disease was found to be 0.16 per 100,000 in the population ^12^. The aetiology of the disease is unknown. Various genetic and environmental factors have been implicated in the causation of the disease, but there is no definite evidence. The pathogenesis involves activation of the innate immune system with subsequent cytokine overproduction, especially interleukins IL-1β, IL-18, IL-6, TNF-α, and IFN-γ ^4^ Several infectious agents have been postulated to trigger the disease in genetically predisposed individuals. It has been proposed that the interplay between host genetic factors, autoimmune mechanisms, and antigens could trigger AOSD ^4, 13, 14^. There have been some case reports of AOSD following a COVID-19 infection in patients who had recently recovered from the illness ^15, 16^. However, only one case of AOSD has been reported following vaccination with the mRNA-1273 COVID-19 vaccine (Moderna), and one after ChAdOx1 nCoV-19 (Oxford-AstraZeneca) vaccine ^5, 6^. It is well-known from various studies that COVID-19 infection causes a release of various cytokines and multi-organ failure (secondary hemophagocytic lymphohistiocytosis). The main cytokines with elevated levels in this condition are IL-1 and IL-6, which are the same cytokines that are activated in AOSD, which points towards a similar mechanism of immune activation ^17^. As in AOSD, IL-1 and IL-6 blockade has been successful in the management of patients with severe COVID-19 infection ^18^. The Oxford-AstraZeneca vaccine is based on a replication-incompetent chimpanzee adenovirus vector that expresses the SARS-CoV-2 spike protein ^19^. Many cases of viral infections, including by adenoviruses, have been reported to trigger AOSD ^4^. It is likely that the adenovirus vector used in the vaccine could trigger the same mechanism as that triggered by the native virus. In addition, the high levels of spike protein could have triggered the disease, as is the case for native COVID-19 infections. Whatever the pathogenesis, the strong temporal association between the vaccination and the onset of symptoms made us suspect the vaccine to be the trigger, although it could be mere coincidence. Further studies are needed to prove the causation.

## Conclusions

AOSD is a rare disease that has been associated with environmental triggers in genetically susceptible hosts. Vaccination against COVID-19 could be a potential trigger for its development for which further studies are required.

## Consent

Written informed consent for publication of their clinical details and/or clinical images was obtained from the patient.

## Data availability

All data underlying the results are available as part of the article and no additional source data are required.
