# Supplementary material for: Case Report: Adult Onset Still’s Disease after vaccination against Covid-19
Source: Wellcome Open Res. 2022 Aug 15;6:333. Originally published 2021 Dec 7. [Version 2] doi: 10.12688/wellcomeopenres.17345.2 (PMC9396110; doi:10.12688/wellcomeopenres.17345.2)
Supplement: Peer review history

# Peer review: Reviewer response for version 2

**DOI:** 10.21956/wellcomeopenres.19936.r51988

**Published:** 2022-08-22

**Authors:** Sankha S. Chakrabarti

**Affiliations:** 1 Department of Geriatric Medicine, Institute of Medical Sciences, Banaras Hindu University, Varanasi, Uttar Pradesh, India

Acceptable, but I could not access the supplementary file of causality assessment. Please ensure it is added.

Are enough details provided of any physical examination and diagnostic tests, treatment given and outcomes?

Yes

Is the case presented with sufficient detail to be useful for other practitioners?

Yes

Is sufficient discussion included of the importance of the findings and their relevance to future understanding of disease processes, diagnosis or treatment?

Yes

Is the background of the case’s history and progression described in sufficient detail?

Yes

Reviewer Expertise:

Geriatric pharmacovigilance, Geriatric neuropsychiatry, COVID-19 (vaccines)

I confirm that I have read this submission and believe that I have an appropriate level of expertise to confirm that it is of an acceptable scientific standard.

# Peer review: Reviewer response for version 1

**DOI:** 10.21956/wellcomeopenres.19175.r49106

**Published:** 2022-03-21

**Authors:** Sankha S. Chakrabarti

**Affiliations:** 1 Department of Geriatric Medicine, Institute of Medical Sciences, Banaras Hindu University, Varanasi, Uttar Pradesh, India

This is an interesting and well managed case.

1. Kindly update the case and death numbers in Nepal to March 2022 figures
2. English language editing is much needed to improve clarity, in the Abstract, Introduction and Case Presentation parts.
3. Use the word hepatitis instead of transaminitis.
4. Use bone marrow cytology (or histopathological examination of bone marrow biopsy sample) whichever is suitable, instead of histopathological bone marrow examination.
5. Please mention the consideration made by the authors while starting high dose steroid. What etiology or condition did they have in mind at the time?
6. Along with the autoimmune thrombocytopenia and hepatitis referenced by the authors (refs 10,11) after COVID-19 vaccination, cases of the related condition rheumatoid arthritis after COVID-19 vaccination may also be mentioned as pathogenesis is broadly linked to Still's disease.
7. The authors discuss two main pathogenetic hypotheses. These may be split clearly in the written text for clarity (adenoviral vector induced and inflammatory cytokines induced). The direct toxicity of Spike protein or cross-reactive antibodies may be mentioned as alternative pathogenetic models. Currently, this is a bit mixed up.
8. Causality assessment file as per old version (not new version) WHO scale for adverse events following immunization must be provided as supplementary material and the causality classification done accordingly- Definite or Probable or Possible, etc.
9. Optional but would be good- If possible the authors should mention whether the patient received the second dose also, and the SARS-CoV-2 anti-Spike IgG level in serum. High levels may be linked to adverse events. Even if not done, I would advise authors to perform this on a follow-up visit for their own knowledge. 

Are enough details provided of any physical examination and diagnostic tests, treatment given and outcomes?

Yes

Is the case presented with sufficient detail to be useful for other practitioners?

Yes

Is sufficient discussion included of the importance of the findings and their relevance to future understanding of disease processes, diagnosis or treatment?

Yes

Is the background of the case’s history and progression described in sufficient detail?

Yes

Reviewer Expertise:

Geriatric pharmacovigilance, Geriatric neuropsychiatry, COVID-19 (vaccines)

I confirm that I have read this submission and believe that I have an appropriate level of expertise to confirm that it is of an acceptable scientific standard, however I have significant reservations, as outlined above.

# Response

**Published:** 2022-08-10

**Authors:** Sudeep Adhikari

**Affiliations:** Patan Academy of Health Sciences, Nepal

Dear reviewer,

 Thank you for your constructive comments that have certainly helped improved the manuscript. 

 Below are point by point responses to your comments.

1. Kindly update the case and death numbers in Nepal to March 2022 figures
  1. Answer- Case and death numbers have been updated.
2. English language editing is much needed to improve clarity, in the Abstract, Introduction and Case Presentation parts.
  1. Answer- Revised as per the suggestion.
3. Use the word hepatitis instead of transaminitis.
  1. Answer- Hepatitis has been used.
4. Use bone marrow cytology (or histopathological examination of bone marrow biopsy sample) whichever is suitable, instead of histopathological bone marrow examination.
  1. Answer- Bone marrow cytology has been used.
5. Please mention the consideration made by the authors while starting high dose steroid. What etiology or condition did they have in mind at the time?
  1. Answer- The diagnosis of AOSD as per the Yamaguchi criteria was considered prior to giving high dose steroids.
6. Along with the autoimmune thrombocytopenia and hepatitis referenced by the authors (refs 10,11) after COVID-19 vaccination, cases of the related condition rheumatoid arthritis after COVID-19 vaccination may also be mentioned as pathogenesis is broadly linked to Still's disease.
  1. Answer- Another article describing the development of seropositive RA has been added in the reference 12.
7. The authors discuss two main pathogenetic hypotheses. These may be split clearly in the written text for clarity (adenoviral vector induced and inflammatory cytokines induced). The direct toxicity of Spike protein or cross-reactive antibodies may be mentioned as alternative pathogenetic models. Currently, this is a bit mixed up.
  1. Answer- Revised as per the suggestion.
8. Causality assessment file as per old version (not new version) WHO scale for adverse events following immunization must be provided as supplementary material and the causality classification done accordingly- Definite or Probable or Possible, etc.
  1. Answer- Causality assessment file has been added as supplementary material.
9. Optional but would be good- If possible the authors should mention whether the patient received the second dose also, and the SARS-CoV-2 anti-Spike IgG level in serum. High levels may be linked to adverse events. Even if not done, I would advise authors to perform this on a follow-up visit for their own knowledge. 
  1. Answer- The patient did not receive the second dose of the vaccine. And the serum level of the anti-spike protein IgG was not performed as it was unavailable.

# Peer review: Reviewer response for version 1

**DOI:** 10.21956/wellcomeopenres.19175.r48155

**Published:** 2022-01-31

**Authors:** Amita Aggarwal

**Affiliations:** 1 Department of Clinical Immunology and Rheumatology, Sanjay Gandhi Postgraduate Institute of Medical Sciences, Lucknow, Uttar Pradesh, India

The authors describe a case of AOSD following COVID-19 vaccination. Though rheumatic disease flare is a known complication of vaccination, development of new disease is less common. AOSD has been reported earlier with COVID-19 as well as with vaccination. 

 This case highlights that if you have persistent fever, leucocytosis thrombocytosis rash and arthritis and no infectious agent or autoantibodies are negative AOSD should be considered.

 AOSD is a diagnosis of exclusion thus it should be emphasized that common diseases must be excluded before prescribing steroids.

Are enough details provided of any physical examination and diagnostic tests, treatment given and outcomes?

Yes

Is the case presented with sufficient detail to be useful for other practitioners?

Yes

Is sufficient discussion included of the importance of the findings and their relevance to future understanding of disease processes, diagnosis or treatment?

Yes

Is the background of the case’s history and progression described in sufficient detail?

Yes

Reviewer Expertise:

Rheumatology

I confirm that I have read this submission and believe that I have an appropriate level of expertise to confirm that it is of an acceptable scientific standard.

# Response

**Published:** 2022-08-10

**Authors:** Sudeep Adhikari

**Affiliations:** Patan Academy of Health Sciences, Nepal

Dear Reviewer,

 Thank you for your feedback to this manuscript.
